# Supplementary material for: Are carbon emissions trading and green financial instruments synergistic? -Comprehensive quantitative research based on content analysis
Source: PLoS One. 2024 Mar 7;19(3):e0298601. doi: 10.1371/journal.pone.0298601 (PMC10919601; doi:10.1371/journal.pone.0298601)
Supplement: S1 Data — (ZIP) [file pone.0298601.s001.zip › Data/地级市Z_F工作报告.zip!/║┌┴·╜¡/╞▀╠¿║╙╩╨/└┤╫╘╣1⁄2╓┌║┼í╛┬φ┐╦╩2╛▌═°í┐_2017.docx]

各位代表：

　　现在，我代表市人民政府，向大会报告工作，请予审议，并请各位政协委员和列席人员提出意见。

　　一、过去五年和2016 年工作回顾

　　过去五年，是我市发展历程中极不平凡的五年。2012 年以来，受煤焦市场持续低迷影响和单一煤炭经济结构制约，全市经济一度连续18 个月负增长。面对严峻形势和多重困难挑战，全市人民迎难而上，知难而进，在省委、省政府和市委的正确领导下，在市人大、市政协的监督和支持下，奋力抵御煤焦市场急转直下的剧烈冲击，竭力克服财税锐减、安全稳定压力增大等诸多不利因素叠加影响，全力聚焦经济建设，努力推动转型发展，全市经济社会发展取得新的进展。

　　五年来，我们攻坚克难全力稳增长，经济实现止跌企稳。自2014 年第三季度实现“由负转正”以来，全市经济始终保持企稳回升态势。初步统计，2016 年地区生产总值（GDP）实现215 亿元，按可比价格计算年均增长1.7%。万元GDP 能耗年均下降4.0%。累计完成全社会固定资产投资610 亿元，实现外贸进出口总额1.5 亿美元。社会消费品零售总额实现100 亿元，年均增长9.5%。物价涨幅保持在2%以下。就业形势总体稳定，累计新增城镇就业5.24万人、再就业3.8万人，城镇登记失业率控制在4.5%以内。地方财政支出累计实现371 亿元，年均增长9.8%，保证了正常运转和重点支出。城镇和农村居民人均可支配收入分别达到21940 元、11310 元，年均增长6.0%和9.5%，分别高于经济增长水平4.3 个百分点和7.8 个百分点。

　　五年来，我们抢抓机遇加快调结构，转型发展迈出新步伐。牢牢把握国家振兴东北战略机遇，积极改造提升传统产业，大力培育新兴产业，经济增长由工业主导转向三次产业协同发展，三次产业比例由2011 年的9.8：60.2：30 调整到2016 年的14.1：36.8：49.1。

　　农业综合生产能力稳步提升，粮食产量达到18.5 亿斤，实现“十三连丰”。累计投入11.2 亿元完善园区基础设施，经济开发区、煤化工园区、“城市矿产”园区、勃利开发区、金沙健康产业园等园区承载能力进一步增强，推进千万元以上产业项目137 个，累计完成投资203.5 亿元。宝泰隆稳定轻烃、隆鹏清洁化学品、吉伟液化天然气（LNG）等煤化工项目，万通管业、晟斯凯特高铁轨枕、乐宝豆沙、天泰玉米、兴发生物质发电等新型建材、绿色食品和新能源项目，奥瑞德蓝宝石、德彩光电、泰安抑爆等高新技术项目相继建成投产或即将投产。中国供销商贸博览城、泓信物流、万通物流、泛东北亚大宗商品国际交易中心投入运营。电子商务发展迅速，电商企业发展到115家，线上年交易额超过3亿元。

　　五年来，我们深化改革努力谋创新，发展活力显著增强。坚持向改革要动力，向创新要效益。把深化“简政放权、放管结合、优化服务”改革放在突出位置，先后7轮清理行政审批事项，市本级保留审批事项256项，减少审批前置件408个，非行政许可全部清零，审批时限压缩53%；建立政府权力清单和责任清单制度，行政权力减少40%；完成“五证合一”“一照一码”“两证整合”登记制度改革，新增企业3296 户、个体工商户26168 户，各类市场主体比五年前增长32%。

　　实施新一轮市区分税制改革，完成金沙新区、经济开发区财政财务管理体制改革，合理划分市区两级事权和财权，在全省率先实现区级财政国库由集中核算向集中支付转轨。规范政府举债融资机制，通过资产证券化等方式融资81亿元，政府债务分类纳入预算管理，偿还政府债务61亿元。农业综合配套改革加快推进，全面开展土地确权登记改革，积极推进农村集体产权制度改革试点、土地承包经营权抵押担保试点。生态文明、司法体制等领域改革取得阶段性成效。

　　五年来，我们全心全意倾力保民生，人民福祉全面改善。实施重点民生工程三年行动计划，困难时期民生投入力度不减，财政累计民生支出260亿元，占公共财政支出比重超过70%。投资10.26亿元开工建设城市供水工程，竣工后将从根本上解决城市水源水质问题。

　　投资80亿元完成中央下放地方煤矿棚户区改造工程，建设北岸新城、如意家园5个居住区，新建住宅4.9万套，4.2万户煤矿棚户区居民搬入新居。采煤沉陷区棚户区项目投资38亿元，22109户棚户区居民将通过新建住房和自主购房形式，彻底改善居住环境。建设福泽家园等城市棚改住房8690 套，新建廉租房、公租房、经济适用房7215套，改造农村泥草（危）房2.1 万户，实施“暖房子”工程260 万平方米。社会保障体系不断完善，社会保险覆盖面不断扩大，社保待遇逐年提高。城乡低保实现应保尽保，建立失能老人护理补贴、困难残疾人生活补贴、重度残疾人护理补贴制度。新建改建一批养老机构，养老事业和养老产业快速发展。脱贫工作全面推进，实施38个“整村推进”项目，1.72万户4.12万农村贫困人口实现脱贫。解决36个村屯、2.3万人的饮水安全问题。投入12.5亿元改善教育设施设备。完成义务教育学校标准化建设，新建改建宏伟、岚峰等中小学校18所，三区全部通过国家义务教育均衡发展评估验收；新建市一中，高中办学规模和水平得到较大提升，重点本科入段率由9.3%提高到22.5%；新建市实验幼儿园及一批县区和乡镇幼儿园，公办幼儿园由5所增加到43所。卫生事业投入资金4.6亿元，迁建市中医院、市妇幼保健院、市精神卫生专科医院，市人民医院外科综合楼竣工投入使用，建设了一批乡镇卫生院、村卫生所和社区卫生服务中心，建立基本药物制度，公立医院全部取消药品加成，医疗卫生服务体系不断完善。市体育中心建成投入使用，文化中心开工建设，城乡硬化广场体育健身设施实现全覆盖。群众性体育活动蓬勃开展，短道速滑等竞技体育成绩突出，五年来共获省级以上金牌160余枚，短道速滑世界冠军增至10位。加强“平安煤城”建设，严厉打击各类违法犯罪，成功侦破“D16”“2·26”“9·8”等重特大案件，织密扎牢社会治安防控网，群众安全感不断增强。进一步健全安全生产责任体系，创新监管手段，强化救援能力，持续开展“打非治违”，关闭矿井80处，安全形势总体稳定。积极回应群众诉求，建立完善信访事项综合评议等长效机制，集中开展信访积案化解专项行动，妥善解决了一大批疑难信访问题。

　　五年来，我们科学规划精心抓建设，城市更加宜居美丽。完成桃山水库二期征地移民，达到蓄水条件；汪清水库消险增容扩建工程竣工，开辟了城市备用水源。新建改造城市西环路、七桦路、大同街东延、松涛路等城市道路14条，路网布局更加畅通合理。全面接收七矿公司“两供一业”，新建、改造供水管网32 公里、供热管网70 公里。新建市第一第二污水处理厂、勃利镇污水处理厂，完成茄子河镇和新强矿生活污水截污工程，全市污水日处理能力达到11 万吨，生活污水处理率达到85%；污泥处置场、垃圾焚烧发电项目试运行。

　　建立了桃山湖国家湿地公园、倭肯河省级自然保护区，高标准建设南北出口、万宝湖坝下滨水景观等园林绿化工程，市民百米见绿千米进园，人均公园绿地面积达到12.3 平方米，成为国家园林城市。大力发展城市公共交通，新建公交调度指挥中心、北岸新城、金沙新区三处公交场站和公路客运总站，新增新型公交车272辆，城市公交覆盖率达80%。全面实施清洁空气行动计划，重点开展7个方面23项整治任务，搬迁治理矸石山6座，淘汰、改造小锅炉373台，淘汰老旧黄标车1.57万辆，燃煤电厂全部完成除尘、脱硫、脱硝改造。对红鲜村等8个村屯进行整村搬迁，铺修农村道路380公里，中心村主街路硬化率、绿化率、亮化率达100%，村容村貌发生巨大变化。

　　五年来，我们持之以恒全面转作风，政府自身建设不断加强。

　　深入开展党的群众路线教育实践活动、“三严三实”专题教育和“两学一做”学习教育，坚持不懈纠正“四风”，持续开展“庸懒散浮”“不作为、慢作为、乱作为”专项整治，政府系统党风政风根本好转。严格落实国务院“约法三章”，三公经费缩减33%。积极推进机构改革，加快职能转变，整合组建卫计委、市场监管局、食药监局、煤管局等部门。稳妥实施县以下机关公务员职务与职级并行制度。优化政务服务，开辟项目审批“绿色通道”，成立企业投诉服务中心，网上政务服务中心开通运行，建立“中介超市”，取消“红顶中介”，坚持零距离接触、零关系办事、零利益服务、零障碍入驻，全力服务招商项目和企业发展。支持纪检监察机关执纪问责，查处违法违纪案件1244 件。严格执行市人大及其常委会的各项决议决定，自觉接受市人大及其常委会的法律监督、工作监督和市政协的民主监督，办理人大代表建议293 件、政协委员提案730 件，政府科学决策、民主决策水平进一步提升，依法执政能力显著增强。

　　刚刚过去的2016 年，是“十三五”规划开局之年，也是我市加快转型全面振兴的攻坚之年。过去一年，我们深入贯彻落实中央、省委省政府和市委工作部署，特别是坚决学习贯彻习近平总书记对我省两次重要讲话精神，制定并实施《推动煤城转型发展全面振兴的实施意见》，进一步理清了转型发展思路，明确了转型发展的主攻方向。主要抓了几个方面的大事要事。

　　扎实推进产业项目建设。坚持“五个要发展”，围绕做好“三篇大文章”，大力开展招商引资上项目，加快转方式调结构，提升项目科技含量。全年重点推进产业项目37个，其中亿元以上项目26个，10亿元以上项目8个，非煤项目32个。勃盛焦化改造、泓泰兴18万吨合成氨30万吨尿素等项目顺利推进，宝泰隆石墨烯、升华医疗器械等高科技项目竣工投产。支持宝泰隆设立新能源新材料研发中心，新成立4 家科技型企业，辟建市级科技企业孵化器，与清华大学、哈尔滨工业大学等院校签订战略合作协议。规划建设石墨烯产业园区和生物技术产业园区，七台河高新技术产业开发区获省政府批准，金沙健康产业园区晋升省级示范基地，产业集聚效应正在形成。

　　切实办好重点民生实事。加快建设城市供水工程，完成总工程进度超过80%。湖东家园采煤沉陷区棚改工程5512 套住宅竣工，建立货币化安置平台，签订16607套货币化安置协议。建立弃管楼长效管理机制，设立了应急维修基金。城市西环路竣工通车，金沙新区客运站建成。推进七矿公司供热区域等老旧供热管网改造，完成供水管网测漏修补。按照医养结合模式，建设失能失智老人养护院、残疾人康复中心。建立城乡特困人员救助供养制度。完成特殊教育学校搬迁改造、市实验幼儿园建设工程，开工建设市六小教学楼。积极申办国家短道速滑七台河训练基地，组织首届冬季户外徒步大赛等群众性体育活动。开展城市清洁空气行动、水污染防治行动，清理规范环保违规项目418 个，更新新能源公交车56 辆，淘汰黄标车老旧车1628 辆、燃煤小锅炉109 台，全市空气质量达标天数为299 天。精准识别农村贫困人口16599 户39138 人，逐一落实精准脱贫措施，14660 人实现当年脱贫。有效落实就业创业扶持政策，积极开发就业岗位，全市实现新就业8522 人，城镇登记失业率为4.31%。积极支持七矿公司改革脱困，拿出部分公益岗位安置第一批组织化分流人员1500 人，成立国有性质再就业服务公司接收第二批分流人员3165人，及时将2602名七矿公司困难职工纳入最低生活保障，发放临时救助资金657万元、医疗救助金155万元。

　　稳步推进各项改革任务。推进公共资源交易市场化改革，成立市公共资源交易中心，工程项目招投标、国有土地使用权出让、政府采购等全部纳入平台运行。实施不动产登记制度改革，市不动产登记中心揭牌运行。农村土地确权登记全面推开，集体产权制度改革试点深入推进，设立2000 万元农业产业发展引导资金，撬动金融资金1.4 亿元。改进项目建设和企业发展扶持机制，制定《产业扶持资金实施暂行办法》，对战略性新兴产业、高新技术产业项目予以扶持；制定《中小型工业企业贷款周转金管理办法》，为中小企业担保贷款5 亿多元。推进国家公立医院改革试点，组建10个覆盖城乡的“医联体”。

　　全力优化经济发展环境。坚持开展政府系统专题学习活动，提高干部专业能力和水平，全力打造服务型政府。取消和调整市本级行政权力2131 项，进一步规范权力运行流程。贯彻落实《关于进一步优化全市发展环境的实施意见》，集中开展执法司法不规范、服务效能不高、中介服务不透明、行政效率不高、诚信体系不健全“五个专项整治”，查处破坏发展环境案件64 起，追责95 人。深入实施“企事厂办”“现场办公”等服务机制，落实企业“宁静工作日”制度，严格执行政府公职人员“四个一律不许”纪律要求，着力打造让企业最满意、让群众最满意的服务环境。

　　坚决维护社会和谐稳定。坚持抓好以煤矿为重点的安全生产，进一步强化安全责任体系建设。出重拳开展“打非治违”歼灭战，公告关闭矿井全部关闭到位，从根本上扭转煤矿安全生产被动局面。全面开展危险化学品、非煤矿山、道路交通、建筑工地、特种设备、消防森防等安全专项治理行动。坚持“四个最严”强化“四有两责”，加大食品、药品等市场监管力度，确保百姓“舌尖上的安全”。严厉打击各类违法犯罪活动，持续开展武装巡逻和网格化巡逻，社会治安秩序平稳。开展依法治访和化解积案两个集中攻坚专项行动，信访案件办结率98.2%，省交办信访积案化解率100%。

　　各位代表，五年来我们的事业在困境中砥砺前行，成绩来之不易。实践证明：只有始终坚持党的领导，自觉维护党中央权威，坚定不移地贯彻中央、省委省政府和市委的决策部署，不折不扣地落实好市委确定的发展目标和任务，才能保持正确的发展方向，不断取得事业新进步。只有始终坚持发展第一要务，紧紧扭住经济建设这一中心，更加注重转方式调结构，更加注重资源节约和环境保护，更加注重改善经济发展环境，才能不断提高发展的质量、效益和水平。只有始终坚持改革创新，不断解放思想、革新理念、创新思路、转换机制，立足发展实际，遵循市场规律，创造性地开展工作，才能不断汇聚和释放转型发展的新活力、新动力、新优势。只有始终坚持勤政为民，把改善民生作为一切工作的出发点和落脚点，做到发展为了人民、发展依靠人民、发展成果由人民共享，才能不断增加人民群众的获得感和幸福感。只有始终坚持法治思维，做到依法行政、科学施政、廉洁从政，建设权责法定、执法严明、公开公正、廉洁高效、守法诚信的法治政府，才能为转型发展提供坚实保障。只有始终坚持责任担当，打造具有强烈使命意识和优良作风的干部队伍，时刻保持昂扬向上、拼搏进取的精神状态和创业激情，才能攻坚克难，不断开创转型发展新局面。

　　各位代表，过去五年取得的成绩，得益于市委的坚强领导、科学决策，得益于市人大、市政协的有效监督、全力支持，得益于各级各部门的务实担当、敬业奉献，得益于全市广大干部群众的齐心协力、顽强拼搏！在此，我代表市人民政府，向各位人大代表、政协委员，向真诚理解、鼎力支持我们的老领导、老同志，向各民主党派、工商联和无党派人士、各人民团体，向所有支持七台河建设与发展的社会各界友人，向在各行各业辛勤劳动的全体市民，表示衷心的感谢！

　　五年来的发展实践，我们更加清醒地认识到，我市经济社会发展仍然面临不少困难和问题，资源性、结构性、体制性矛盾仍很突出。经济发展仍未摆脱高度依赖资源的发展惯性，经济发展新动能不足，战略性新兴产业的培育和发展不快，单一产业结构抵御市场风险能力不强。城市基础设施和公共服务设施不完善，特别是城市地下网管系统老化严重，大气、水等环境污染问题还很突出，城市承载和公共服务能力与市民日益增长的需求仍有差距。　“12·1”“11·29”等煤矿事故充分暴露安全生产基础仍很薄弱，加之社会矛盾凸显，保持社会和谐稳定仍面临巨大压力。一些干部精神不振、担当不够，不愿为、不敢为、不会为，政府职能转变还不能很好适应新形势新要求。这些问题，既有发展中客观规律性的矛盾，也有我们工作方法、态度和作风上的问题，在今后的工作中我们必须高度重视，并采取得力措施认真解决。

　　二、未来五年发展目标和主要任务

　　未来五年，是我市全面建成小康社会的决胜阶段，是全面实施“十三五”规划、实现转型振兴的关键时期。刚刚闭幕的市第九次党代会以“激发内生动力、加快转型发展，奋力走出煤城全面振兴的新路子”为主题，谋划了今后五年的奋斗目标，绘就了七台河未来发展的宏伟蓝图，为政府工作指明了方向。今后五年，我们既要看到外部环境复杂多变、面临诸多困难和挑战，也要看到党的十九大将引领我国进入新的发展阶段，看到国家振兴东北、支持资源型城市转型发展难得的历史机遇，还要看到我们多年积累的转型发展潜能正在逐渐释放。机遇承载使命，挑战考验担当，习近平总书记系列重要讲话特别是对我省两次重要讲话精神，为我们推进各项事业指明了前进方向，注入了强大动力。我们要按照统筹推进“五位一体”总体布局和协调推进“四个全面”战略布局的总要求，努力践行“创新、协调、绿色、开放、共享”新发展理念，认真落实省委实现龙江全面振兴和市委推动煤城转型发展的总体部署，把转型振兴作为第一要务，把改革创新作为第一动力，把民生福祉作为第一追求，把生态建设作为第一责任，把安全稳定作为第一前提，把从严治党作为第一保障，不断激发内生动力，加快推进煤城转型发展全面振兴。

　　今后五年，经济社会发展的主要预期目标是：地区生产总值（GDP）年均增长6.5%以上，一般公共预算收入年均增长5%以上，固定资产投资年均增长7%以上，城镇和农村居民人均可支配收入年均分别增长7.1%、7.8%以上，万元GDP 能耗降低15%。这些目标的确定，既与“十三五”规划和“第一个百年”奋斗目标相衔接，又充分体现市第九次党代会提出的新要求。我们要全力以赴完成既定目标，努力实现更好更快发展。

　　——加快推进转型发展。突出“无中生有”扩增量、“有中生新”提存量，加快转变过度依赖资源消耗的发展方式，加快调整以煤为主的产业结构，大力发展煤炭精深加工、新材料新能源、生物和医药、先进制造、绿色食品、现代服务业等六大重点产业，基本建立起技术先进、结构优化、竞争力强的多元化现代产业体系。

　　——深入释放创新动能。实施创新驱动发展战略，以新理念培育发展新技术、新产业、新业态、新商业模式，加速转化创新成果，集聚更多的高端产业、高新技术、高级人才，形成具有资源型城市转型发展鲜明特色的科技创新体系，培育发展一批高科技产业集群，提升高新技术产业在规模以上工业中比重。进一步完善有利于推动创新发展的体制机制，全面融入“龙江丝路带”建设，不断增强煤城发展活力、内生动力和整体竞争力。

　　——打造宜居美丽家园。深入推进以人为本的新型城镇化，优化城镇空间布局，加快小城镇建设。进一步完善基础设施和公共服务设施，不断增强城市服务保障能力，形成节约资源和保护环境的空间格局、产业结构、生产生活方式，让煤城的天更蓝、山更绿、水更清，建设独具特色、绿色生态、自然和谐的美丽宜居滨水城市。

　　——提高市民幸福指数。突出人的全面发展，实现好、维护好、发展好最广大人民的根本利益。采取积极有效措施促进稳定充分就业，进一步完善社会保障体系，推进基本公共服务更趋均等化，教育、医疗、卫生等服务能力和水平明显提升，社会公平正义得到有效维护，努力使发展成果更多更公平地惠及全市人民。

　　三、2017年重点工作

　　2017 年是新一届政府履职尽责的开局之年。做好政府工作，必须全面贯彻党的十八大和十八届三中、四中、五中、六中全会精神，深入学习贯彻习近平总书记系列重要讲话特别是对我省两次重要讲话精神，认真落实中央、省委省政府和市委决策部署，坚持稳中求进总基调，坚持以提高发展质量和效益为中心，坚持以推动转型发展为主线，坚持以振兴实体经济为基石，突出创新驱动，激发内生动力，围绕“五个要发展”做好“三篇大文章”，进一步提升传统比较优势，加快培育新的发展动能，不断深化改革扩大开放，更加注重改善民生，更加注重生态建设，更加注重和谐稳定，努力走出新形势下转型发展全面振兴的新路子。

　　主要预期目标是：地区生产总值（GDP）增长6.0%—6.5%，一般公共预算收入增长3%左右，固定资产投资增长10%左右，社会消费品零售总额增长6%左右，城镇登记失业率控制在4.5%以内，城乡居民收入增长高于经济增长。确定地区生产总值增长6.0%—6.5%，留有0.5%的弹性空间，体现坚持稳中求进的总要求，既与“十三五”规划目标相契合，又与省委经济工作会议要求相一致。

　　综合分析各方面情况，2017 年我们面临的形势仍错综复杂。世界经济深度调整、复苏缓慢，国内经济下行压力依然存在，产能过剩和需求结构升级的矛盾仍很突出。同时也要看到，国家推动东北振兴、资源型城市转型系列支持政策力度空前，我省深入推进“五大规划”，建设“龙江丝路带”，特别是习近平总书记两次针对黑龙江发展的重要讲话，为我市转型发展提供了新的政策机遇和强大发展动力。国家深入推进供给侧结构性改革，推动“三去一降一补”五大任务取得实质进展，继续化解钢铁、煤炭行业过剩产能，煤焦市场供需形势趋于平稳，将为我们做好2017 年各项工作创造较为有利的条件。我市经济“由负转正”以来，始终保持稳步回升态势，为实现今年的目标任务奠定了良好基础，产业结构的调整优化、重点产业项目的发力见效、新动能的培育生成，也将支撑经济进一步回升向好。只要我们扬长避短、扬长克短、扬长补短，保持定力，攻坚克难，就一定能够实现全年经济社会发展目标。

　　今年要重点做好以下九个方面的工作。

　　（一）创新驱动转型升级，加快构建现代产业体系

　　加快转型发展，创新是核心驱动，项目是根本支撑。坚持把创新作为引领发展的第一动力，把产业项目建设作为“一号工程”，加大招商引资上项目力度，编制实施《七台河资源型城市产业转型攻坚行动计划》，加快构建支撑转型发展的现代产业体系。重点推进五千万元以上产业项目34 个，总投资176.2 亿元，年度投资47.4 亿元。其中非煤项目30个，总投资99.2亿元，年度投资23.2亿元。

　　加快传统产业转型升级。注重引入新技术、新工艺，开发新产品，提升传统产业竞争力。做强做大“煤头化尾”，加快煤炭资源勘查勘探，推进煤矿改造、整合、重组，加强煤矿标准化建设，合理有序谋划开发建设新矿井；大力发展现代煤化工产业，加快宝泰隆30万吨稳定轻烃、隆鹏清洁化学品项目建设，推进勃盛、泓泰兴焦化升级改造项目建成投产。做大做精“粮头食尾”，推进金晖食品方便米饭、利健生物蓝靛果、宏泰松果松仁系列产品、伊鑫清真食品等项目，壮大绿色食品产业。深度开发“城市矿产”，完善和规范再生资源回收体系，推进康泰公司废旧钢铁分拣加工、矿山设备拆解和再制造、废弃电子电器拆解等项目尽快投产。

　　大力培育战略性新兴产业。加快发展高端石墨新材料产业。重点推进宝泰隆5 万吨电池负极材料项目、东润矿业12 万吨石墨精粉选矿项目。依托清华大学—宝泰隆石墨烯研究院，加快石墨烯应用领域成果转化，积极引进产业化项目。推进与哈宜公司、琅琊台集团、阜丰集团等企业合作，开工建设琅琊台集团年处理30万吨玉米淀粉加工项目、5 万吨衣康酸项目、30 万吨秸秆生物饲料项目。以奥瑞德视窗材料、德彩光电显示屏等项目为带动，积极引进彩虹集团、通普科技等高新技术企业，开发半导体照明（LED）器件、组件、软件等全产业链光电产业项目。紧紧抓住中国兵装集团在哈建立福克斯整车生产线契机，积极引进汽车配件生产企业，扩大农业机械、节能设备、矿山设备、消防设备等生产规模，加快制造业提档升级。支持双叶家具适应消费个性化需求趋势，实施生产线柔性改造，提升家具定制化、智能化制造水平。

　　提升园区平台承载能力。按照“一区多园”模式，加快推进省级高新区建设，搭建高新技术产业集聚发展平台，积极引进一批高成长性、高科技含量、高附加值项目，提升产业承载能力，争创国家高新区。集中力量抓好石墨新材料产业园区、生物技术产业园区建设，开工建设生物技术产业园供水管线、自备电站、污水处理厂，谋划建设公铁物流中心，促进新材料产业、生物技术产业集聚发展，打造新的经济增长极。加快“城市矿产”园区污水处理厂、固废中心、供热中心等配套设施建设，完成七矿公司火药库迁建，完善项目承载条件。继续加强勃利开发区、金沙健康产业园建设。深化园区管理体制改革，探索“园区+平台”的运营管理模式，推进园区规模扩张、发展提速、转型升级。

　　创新项目实施推进机制。把招商引资作为上项目、扩总量、增税收、促就业的重要抓手，围绕“六大重点产业”，注重从产业链、价值链上谋划储备一批市场前景好、带动能力强的大项目，加强与珠三角、长三角、京津冀、环渤海等地区对接合作，瞄准行业龙头企业、央企、500 强民营企业等深入开展专业化精准招商。引入社会资本扩大产业扶持基金规模，完善管理运营方式，制定鼓励投资产业目录，提高资金扶持效能。严格落实市、县（区）两级领导产业项目建设“四帮四保”责任，全面营造安商、富商良好营商环境。

　　（二）优化盘活存量资源，增强实体经济活力

　　在优化工业存量上下功夫，抓好现有企业发展，通过对存量企业的挖潜、盘活、重组、优化，全面提升实体经济活力，把旧存量转化为新增量。

　　积极盘活存量企业。全力支持七矿公司改革脱困、转型发展，推进“三项制度”改革，加快辅业市场化改革，发展电网经营、机械加工、工程建筑、物流仓储等非煤产业，努力增强企业内生动力，尽快走上扭亏脱困、良性发展轨道。对停产半停产企业，引导帮助做好市场对接，引进投资重组盘活一批、升级改造提升一批，促进老企业焕发新生机。坚决清理“僵尸”企业和“半截”项目，创造条件推进企业兼并重组，有效盘活“沉睡”资产，“腾笼换鸟”承载新项目。

　　优化生产要素配置。稳步推进电力改革，积极开展大用户与发电企业直接交易，降低企业用电成本。加快庆—鸡—林输电线路工程、茄子河输变电工程建设进度，提高电力输出和保障能力。定期召开银企对接会，扩大工业企业贷款周转金使用范围，帮助企业缓解资金紧张问题。鼓励企业通过“新三板”挂牌、发行债券等方式，到资本市场直接融资。继续实行企业外购原料“绿色通道”，积极帮助协调解决产品外运。

　　加快发展中小微企业。建立小微企业创业园，大力发展专业化众创空间和科技企业孵化器，支持鼓励大众创业、万众创新。出台《扶持小微企业发展的意见》，设立1000 万元小微企业贷款担保金，制定相关财税金融扶持政策，扶持个体工商户、小微企业“个转企”“小升规”。落实“千户科技型企业三年行动计划”，全力扶持打造一批科技“小巨人”企业。

　　（三）推进农业供给侧结构性改革，大力发展绿色有机农业

　　决胜小康，富裕农民是关键。围绕农民增收、脱贫致富，大力发展具有鲜明供给优势的绿色有机农业，促进农林牧渔结合、种养加销一体、一二三产业融合发展，走科技支撑、资源节约、产出高效、生态有机的现代农业发展之路。推进小城镇和美丽乡村建设，统筹城乡发展。

　　加快种植结构调整。推动粮经饲统筹发展，引导农民减玉米、稳定并适当增加水稻、增加大豆杂粮、扩草蓿和果蔬，扩大绿特色作物种植面积。坚持市场需求导向，进一步明确绿色有机主攻方向，重点发展红小豆、谷子、芽豆等杂粮产业，万寿菊、白瓜子、甜叶菊等经济作物产业，高钙菜、黑甜甜葡萄、小毛葱等果蔬产业，蓝靛果、红松果、黑木耳、北药等林下经济产业，特色农业面积达40 万亩。实施黑土地保护工程，加强生态高产标准农田建设，推进勃利县水土流失治理、大泥鳅河堤防等重点工程。推进农业“三减”行动计划，复制推广“鸭稻”“鱼稻”“鹅玉米”等生产模式，扩大“三品一标”覆盖范围，全面提升农产品品质。

　　大力发展现代畜牧业。精准落实畜牧贷款贴息、规模肉羊场良种补贴、全株青贮玉米补贴、可繁母牛扩群增量补贴等扶持政策，大力发展标准化规模养殖，加快亿丰、鹏飞肉牛繁殖标准化养殖基地项目建设，推进中航卓信全产业链生猪养殖项目。加大畜禽品种改良工作力度，不断提高良种覆盖率。大力推广畜牧兽医先进适用技术，完善畜牧技术和信息服务体系。

　　提升农业产业化水平。以“农头工尾”为抓手，强化龙头企业带动，推进金穗30 万吨稻谷、兴粮30 万吨玉米、天泰鲜食玉米等粮食深加工项目，以及中利科技万寿菊黄体素深加工等农业产业化项目建设。探索农业农村领域创新创业模式，培育建立“星创天地”，促进科技兴农富农。抓好省级“互联网+农业”示范基地建设，完善食品质量追溯体系，借助“绿博会”“绿色食品旗舰店”“地方特色馆”等平台，扩大“东北老勃”“三胜杂粮”等绿、特、名地域品牌市场影响力，实现“种得好”向“卖得好”转变。推进合作社规范化建设，引导鼓励发展联合社，提高合作层次和水平，建立生产、经营、服务整合发展的复合型农业经营体系。

　　坚决打赢脱贫攻坚战。坚持精准扶贫、精准脱贫，加大教育脱贫、转移就业脱贫、医疗保障脱贫、政策兜底脱贫等精准扶贫措施落实力度。把发展产业作为脱贫攻坚的重中之重，推进“一村一品”产业扶贫，注重示范带动，扶持贫困村发展“菌、果、蔬、禽、畜”等特色种养业。加大财政投入力度，吸引社会资本参与扶贫开发，加快贫困村基础设施和公共服务设施建设。确保今年勃利县省级贫困县脱贫摘帽，2018年现行标准下农村贫困人口全部脱贫。

　　建设美丽宜居乡村。加强小城镇、林场和农村人居环境治理，完善水、电、路、网络等基础设施，推进垃圾粪污无害化处理，提高公共服务水平，引导农村人口向小城镇集中。创新和加强农村社会管理，培养新型职业农民，提升农民素质，倡导文明乡风，建设整洁、优美、宜居的新农村。

　　（四）激活潜力补齐短板，加快发展现代服务业

　　按照服务产业发展、扩大消费需求、完善城市功能的要求，大力发展现代服务业，不断拓展服务业发展空间，加快形成新的经济增长点、市场消费点、就业拉动点。

　　大力发展电子商务产业。提高各级电商产业园区的综合服务能力，提高知识产权、金融平台、创业辅导、管理提升、专业培训、包装设计、电商物流等服务运营水平，做大做强自主平台公司，整合建立集配中心、分拣中心，增强园区承载及孵化能力。促进“互联网+”新业态创新，加快发展云计算、大数据、物联网、移动互联网，积极引进蜂巢电商、海仓科技、中建环球等电商平台，促进电子商务与实体经济深度融合，扩大地方名特优产品网上销售份额，打造“龙江东部电商谷”。

　　稳步发展商贸物流业。利用沉陷区治理、棚户区改造腾空土地，规划建设综合物流园区，加快推进中国供销商贸博览城二期、万通与传化合作物流项目一期工程建设，积极引进冷链物流建设项目，努力打造黑龙江东部物流节点城市。完善海关监管场所功能，引入商检职能，推进保税仓库建设，为企业提供货物通关、储存、运输等便利化服务。积极探索电子口岸、跨境电商等内陆经济开放新模式，重点面向俄罗斯、东北亚市场，打造绿色有机农产品、家具家装等产品出口生产加工基地。

　　发展医、养、游相结合产业。以开发区域性特色旅游为方向，重点开发观光农业、休闲养生、赏冰乐雪等项目，按照4A 景区标准完善西大圈国家森林公园旅游道路、游客中心等基础设施，推动勃利森林公园、吉兴河风景区旅游功能点状开发。积极发展“候鸟式养老”产业，推进天赐智慧医养结合养老项目、万景集团石龙山养老养生示范基地项目建设。

　　加快发展新兴服务业。大力发展研发设计、商务服务、金融保险、软件外包、检验检测认证、人力资源开发等生产性服务业，加快发展健康保健、家政服务、休闲健身等生活性服务业，推动生产性服务业向专业化延伸，生活性服务业向高品质转变。

　　（五）规划先行建管并重，建设美丽宜居家园

　　以人的城镇化为核心，增强城市规划的科学性，全面提升城市规划建设管理水平，提高生态环境承载能力，不断改善人居环境。

　　完善城市整体规划布局。加快构建“多规合一”规划体系。市县协同推进高铁站区的规划和开发，加快城区老工业区和独立工矿区搬迁改造，深入治理采煤沉陷区，科学规划利用棚改腾空土地，为城市未来发展预留空间。着眼滨水城市建设，谋划桃山湖整体规划开发，推动“产、城、人、文”融合发展。

　　加强城市建设管理。完成牡佳客运专线征地动迁，结合牡佳高铁站谋划综合交通枢纽项目，推动七密铁路、七密高速和七台河机场的规划论证工作。新建如意家园至桃山区道路、东昌街、大同街东延二期、奋斗路北延工程，完成仙洞山道路、步行街改造。加快供水工程建设，确保如期通水运行。逐步推进综合地下管廊建设，统筹各类管线铺设。继续推进老旧供热管网改造工程，对新接收的七矿公司8 个分散供热区域管网进行集中供热改造。实施生产生活用水分离工程，加大供水管网改造力度，改造老旧住宅内部管网和二次供水设施。续建学府街东段雨污分流工程，新建中心河污水处理站、桃山垃圾场渗滤液处理站，抓好第二污水处理厂、垃圾发电厂、污泥处置厂的协调运营。启动桃山湖坝下倭肯河区段综合整治工程，实施新七台河堤防工程，开展万宝河水体治理。新建学子园绿化工程，推进园林绿化提档升级、补植增量。改善百姓出行条件，新建北岸新城国家一级客运站，改造茄子河客运站，淘汰微型出租车，新增电动公交车100台，加强通村公路管理和养护，推进农村客运班线公交化。加强城市精细化管理，整合公安、交警数字化资源，建设市中心区数字化城市管理工程，增强金沙新区城市管理职能。加大环卫设施装备投入，提高环卫机械化作业水平。

　　加大环境保护力度。深入推进大气污染、水污染防治行动计划，制定实施土壤污染防治工作方案，打好水、气、土污染防治“三大战役”。推进节能减排，推广使用清洁能源，削减主要污染物排放总量。大力整治燃煤污染、工业污染和生活污染，严控秸秆焚烧。继续淘汰老旧车辆，完成黄标车、10蒸吨及以下燃煤锅炉淘汰工作。

　　加强生态保护和修复。推行河长制，强化桃山湖水源地生态保护、倭肯河流域污染防治。保护森林、湿地资源，推进桃山湖国家湿地公园、倭肯河省级湿地自然保护区、那丹哈达岭省级地质公园等生态保护项目建设。

　　（六）守好底线增进福祉，扎实办好民生实事

　　以人民为中心，把增进人民福祉、促进人的全面发展作为一切工作的出发点和落脚点，确保困难时期民生投入不减、力度不变、标准不降，不断提高人民群众生活水平。

　　加快保障性安居工程建设。完成如意家园、湖东家园小区全部住宅工程建设，完善配套设施。加快棚改实物安置，推进货币化安置。完成金沙新居保障房后续工程，增加公租房供给，为人才就业创业提供住所保障。启动七矿公司物业移交范围内住宅修缮工程，推进物业管理市场化移交。

　　促进更加充分就业。建设职业技师学院创业大厦和实验楼，打造全市公共实训基地。加强就业技能培训、岗位技能提升培训和创业培训，落实残疾人、失地农民、“4555”人员等就业困难群体和零就业家庭就业保障政策。鼓励发展“新兴手工”等居家创业项目，通过灵活就业等形式吸纳更多闲散劳动力就业。落实好大学生创业扶持政策，发挥大学生创业种子基金引领作用，鼓励大学生自主创业。做好七矿公司分流人员安置工作，促进分流人员创业就业。深入推进劳动合同制度和劳动用工备案制度，切实维护劳动者合法权益。

　　统筹推进城乡社会保障体系建设。落实全民参保登记计划，积极推进社会保险由制度全覆盖向人群全覆盖。全面实施城乡居民大病保险制度，健全重特大疾病救助制度，扩大城乡医疗救助范围，提高城乡低保保障水平和特困人员救助供养标准，加大低收入群体和残障弱势群体社会救助力度。做好七矿公司困难职工救助工作。推动“三社联动”居家养老试点，加强社区日间照料设施升级改造，为居家老年人提供方便快捷周到的服务。

　　促进教育均衡协调发展。新建中小学综合实践基地、茄子河区双鹤小学，完成市第六小学翻建工程。加快教育信息化建设步伐。勃利县义务教育均衡发展通过国家评估验收，茄子河区、桃山区、新兴区在基本均衡的基础上实现优质均衡。加快发展现代职业教育，提升职业技师学院服务地方经济发展的办学水平。

　　提高城乡居民健康水平。加快市人民医院全科医生规范化临床培训基地、市中医院康复诊疗中心和“治未病”中心建设，推进医疗卫生信息化，完善市、县（区）、乡镇和社区三级网络平台，开展远程医疗合作，实施“百名研究生计划”，提升诊疗救治水平。健全疾病防控体系，加强慢性病综合防控，推进疾病治疗向健康管理转变。

　　加快文体事业发展。完善公共文化服务体系，加快市文化中心项目建设，鼓励具有地域特色的文化艺术创作，扩大和引导文化消费。依托短道速滑竞技体育优势，积极对接北京冬奥会冰上项目，做好“国家短道速滑七台河体育训练基地”申报和工作开展，引领冰雪运动时尚，积极承接大型赛事，推动体育产业发展。

　　（七）坚持问题导向，深化重点领域改革

　　深入贯彻中央改革总体要求，重点推进有利于增添经济发展动力的改革、有利于促进社会公平正义的改革、有利于增强人民群众获得感的改革、有利于调动广大干部群众积极性的改革，汇聚转型发展新动能。

　　深化经济体制改革。推进东北地区民营经济改革试点工作，落实省委省政府关于支持民营经济发展的50 条意见，放宽准入、降低成本、改善服务，引导建立现代企业制度，营造公平竞争环境。推进财税体制改革，完善市区收入分享机制，理顺市区两级财政支出责任。规范公共资源交易中心运行，建立健全交易监督管理机制，经营性公共资源全部采取市场化招标方式公开配置。完善多元化投融资机制，鼓励和支持民营企业、社会资本进入基础设施、公共事业等领域。在交通、城建、环保、医疗、养老等领域推广政府和社会资本合作模式，建立全市PPP 项目库，凡能使用PPP 模式的项目，原则上都要采取PPP 方式实施。

　　推进农业综合配套改革。加快推进农村集体产权制度改革，推动土地所有权、承包权、经营权三权分置，完成土地确权登记。加快区县、乡镇两级土地流转服务平台建设，引导土地经营权健康有序流转。引进战略投资者，推动农信社改制为农商行。健全现代农业信用体系，提高农产品加工企业融资能力。加大农业保险保费补贴力度，推动产量保险、价格保险等试点，防范农业风险。

　　完善文教卫生事业改革。全面实施小学学科考试等级评价制度，适应国家高考改革要求推进教育教学改革。继续深化公立医院改革，完善医联体模式和分级诊疗制度，推进城乡居民基本医疗保险整合。

　　抓好其他改革工作。稳妥推进国有林场改革，加快从事生产经营活动事业单位改革，推动环保机构监测监察执法垂直管理体制改革。

　　（八）标本兼治综合治理，确保社会和谐稳定

　　转型发展，安全稳定是前提。继续推进“平安煤城”建设，健全公共安全体系，确保社会安全稳定，增强人民群众安全感。

　　全力抓好安全生产工作。牢固树立红线意识和底线思维，严格落实安全生产两个“一票否决”制度，加强安全责任体系建设。严格安全准入标准，强化源头治理。加强安全风险管控和隐患排查治理双重体系建设，强化事前、事中和事后监管，建立有力的协调联动机制，消除监管空白，坚决防范和遏制较大以上事故。积极推进安全生产诚信体系建设，完善企业安全生产不良记录“黑名单”制度。突出抓好煤矿安全工作，落实落靠地方煤矿分类监管责任，严厉打击煤矿违法违规建设生产、超能力生产和不安全生产行为，坚决完成地方煤矿关闭任务，整合资源淘汰落后产能。加快东部煤化工救援基地建设，提升重点领域安全事故和突发事件的应急处置能力。强化食品药品风险监控和监督抽检工作，加强食品监管各部门联合执法，保障人民群众饮食用药安全。加强重大区域性火灾隐患整治，抓好危险化学品、道路交通、建筑施工、特种设备、非煤矿山、城镇燃气、烟花爆竹、人员密集场所等各领域的安全工作，确保人民群众生命财产安全。

　　提升社会综合治理能力。实施公安“六大建设工程”三年行动计划，构建立体化社会治安防控体系，完善“平安家园”视频监控系统建设，深化城乡社区警务工作，坚持“五位一体”巡逻联动执勤模式，提高街面见警率，保持打击违法犯罪的高压态势。强化社区矫正工作，加强重点人员管控。

　　维护社会和谐稳定。健全完善信访综合评议机制、社会矛盾预警调处化解机制，集中开展信访积案会战和治理非访攻坚，理顺信访三级结案程序，打造网上信访主渠道，推动诉访分离，合理诉求依法解决。

　　（九）科学执政服务为民，建设人民满意政府

　　面对新形势新任务新要求，牢固树立和贯彻新发展理念，切实转变职能，努力建设人民满意政府。

　　坚持依法行政。严格遵守宪法和法律，运用法治思维和法治方式深化改革、推动发展、化解矛盾、维护稳定，推进各级政府机构、职能、权限、程序、责任法定化。健全依法决策机制，完善公众参与、专家论证、风险评估、合法性审查、集体讨论决定等重大行政决策程序，全面推行政府法律顾问制度。建立行政裁量权基准制度，坚持“双随机、一公开”，严格规范行政执法，加大政务公开的广度和深度，确保权力在法治轨道上正确运行。

　　坚持为民施政。深化简政放权，加快推进“互联网+政务服务”和信息惠民工程，推行行政许可事项、公共服务事项网上办理。加强和规范乡镇、社区便民服务中心建设，扩大便民服务事项范围，做到服务群众“零距离”。坚持“民情恳谈”“企事厂办”等制度，严格执行政府公职人员“四个一律不许”要求，全力优化发展环境，为人民群众提供最满意的服务，为企业提供最满意的服务。

　　坚持务实勤政。强化干部专业思维、专业素养，打造高素质的干部队伍，提升政府工作专业化水平。健全督查问责机制，狠抓重点工作推进落实，不断提高政府执行力。持续用力纠正“四风”，深入开展“庸政、懒政、怠政”“吃拿卡要”及“下梗阻”等专项整治，对不作为、乱作为严肃问责，全力打造务实担当的良好政府形象。

　　坚持从严治政。牢固树立政治意识、大局意识、核心意识、看齐意识，向以习近平同志为核心的党中央看齐、向党的理论和路线方针政策看齐，严守党的政治纪律、政治规矩。强化党风廉政建设，对侵害企业和群众利益问题、腐败问题坚持“零容忍”。严肃查处各级各类违纪违法案件，推动纪检监察工作向基层延伸，确保干部清正、政府清廉、政治清明。

　　各位代表，政府的权力来自人民，必须对人民负责，受人民监督。政府将认真执行人大及其常委会的决议、决定，坚持重大事项向人大报告、向政协通报制度，主动接受人大法律监督、工作监督和政协民主监督，切实做好人大代表建议、意见及政协提案办理工作。认真听取各民主党派、工商联和无党派人士意见，主动接受舆论监督和公众监督，不断改进工作，提高行政质量。

　　各位代表，全力做好2017 年政府工作，对于推动煤城转型发展、全面建成小康社会具有十分重要的意义。空谈误国，实干兴邦。唯有脚踏实地、扎实苦干，才能实现市九次党代会确定的未来五年发展蓝图。让我们更加紧密地团结在以习近平同志为核心的党中央周围，在市委的坚强领导下，凝心聚力，开拓进取，奋力谱写煤城转型振兴的历史新篇章，以优异成绩迎接党的十九大胜利召开!
